# Treatment of Human Lens Epithelium with High Levels of Nanoceria Leads to Reactive Oxygen Species Mediated Apoptosis

**DOI:** 10.3390/molecules25030441

**Published:** 2020-01-21

**Authors:** Belal I. Hanafy, Gareth W. V. Cave, Yvonne Barnett, Barbara Pierscionek

**Affiliations:** 1 School of Science and Technology, Nottingham Trent University, Clifton Lane, Nottingham NG11 8NS, UK; belal.hanafy2017@my.ntu.ac.uk (B.I.H.); gareth.cave@ntu.ac.uk (G.W.V.C.); Yvonne.barnett@anglia.ac.uk (Y.B.); 2 Faculty of Science and Technology, Anglia Ruskin University, East Road, Cambridgeshire CB1 1PT, UK; 3 School of Life Science and Education, Staffordshire University College Road, Stafford ST4 2DE, UK

**Keywords:** apoptosis, genotoxicity, reactive oxygen species, cerium oxide, mitochondria

## Abstract

Nanoceria (cerium oxide nanoparticles) have been shown to protect human lens epithelial cells (HLECs) from oxidative stress when used at low concentrations. However, there is a lack of understanding about the mechanism of the cytotoxic and genotoxic effects of nanoceria when used at higher concentrations. Here, we investigated the impact of 24-hour exposure to nanoceria in HLECs. Nanoceria’s effects on basal reactive oxygen species (ROS), mitochondrial morphology, membrane potential, ATP, genotoxicity, caspase activation and apoptotic hallmarks were investigated. Scanning electron microscopy-energy dispersive X-ray spectroscopy (SEM-EDX) studies on isolated mitochondria revealed significant uptake and localization of nanoceria in the mitochondria. At high nanoceria concentrations (400 µg mL^−1^), intracellular levels of ROS were increased and the HLECs exhibited classical hallmarks of apoptosis. These findings concur with the cells maintaining normal ATP levels necessary to execute the apoptotic process. These results highlight the need for nanoceria dose-effect studies on a range of cells and tissues to identify therapeutic concentrations in vitro or in vivo.

## 1. Introduction

Nanoparticles offer great potential as medical devices due to their unique physicochemical properties [1,2,3]. For example, cerium oxide nanoparticles “nanoceria” are extensively researched for biomedical and drug delivery applications because of their unique recyclable antioxidant, neuroprotective, radioprotective and anti-inflammatory properties [4,5,6,7,8]. These properties arise from the ability of cerium ions to co-exist and transition between trivalent and tetravalent oxidation states (Ce^3+^, Ce^4+^) at the surface defects present in the CeO_2_ crystalline structure allowing nanoceria to release or acquire oxygen depending on the ambient environment [9,10]. Additionally, nanoceria are increasingly used for industrial applications such as an additive to diesel fuel (commercially available under several trade names such as Envirox™) to reduce soot emissions and decrease fuel consumption [11,12]. This has led to increased exposure to nanoceria which warrants investigation of their toxicological effects and interactions with various eukaryotic cells. As such, nanoceria was considered a high priority for toxicological evaluation [12,13].

Despite the exponential rise in the use of new nanomaterials, nanotoxicological evaluation is not carried out with a similarly high rate [14]. Hence, assessing and evaluating the biocompatibility of nanomaterials are crucial issues for both the public and the scientific community to enable informed decisions to be made regarding their use. Various studies have focused on the toxicity mechanism of different types of nanoparticles, such as Ag, Zn, and Si, in different cell lines in investigations of oxidative stress as a cause of toxicity [3]. Cerium oxide nanoparticles are unique as they possess inherent antioxidant properties and hence, studying their ability to induce oxidative stress presents a novel and crucial perspective on their potential function and application. The toxicity of nanoceria has been studied in various in vivo and in vitro models and more investigations into the mechanism of their action are needed [13]. Additionally, many of the toxicological studies are performed on nanoceria with unknown or poor colloidal stability, a common problem for nanoceria, which gives rise to conflicting toxicological profiles. Although nanoceria are known for their powerful antioxidant properties, some studies have reported their ability to cause oxidative stress [13,15,16]. Ocular exposure is of particular interest due to the ease for sustained environmental exposure. Although nanoceria have shown promise in treating some ocular diseases such as retinal degeneration [17], corneal inflammation [18], glaucoma [19], and cataracts [20], there is a paucity of knowledge about toxicity mechanism of nanoceria in any ocular tissue. The human lens epithelial cell line (HLEC) provides a suitable model for studying toxicity of nanoceria on the eye lens, as these cells are the lenticular stem cells from which the lens fiber cells differentiate. Damage to the lens epithelium disrupts protein expression and could lead to aggregation of proteins that causes light scatter and manifests as cataract [21].

Recently, it has been shown that highly stable and well characterized monodisperse cerium oxide nanoparticles (4 nm) coated with ethylene glycol and its acetate derivatives, ie ethylene glycol coated nanoparticles (EGCNPs) can protect HLECs from oxidative stress when used in concentrations as low as 50 µg mL^−1^, and the nanoparticles were non-cytotoxic up to 200 µg mL^−1^ [20]. When the concentration of nanoceria was increased to 400 µg mL^−1^, a decrease in viability, measured by MTT assay, was observed. However, the cells were still able to proliferate normally for over three days without signs of necrotic damage [20]. This suggest that the nanoparticles at this concentration could interfere with mitochondrial functions and this has warranted further investigation.

In this study, we investigated the mechanism of toxicity caused by short-term exposure to different doses of EGCNPs. Here, we report the effect of different EGCNPs concentrations on various cellular parameters. The results show that although nanoceria have antioxidant properties at low doses (< 200 µg mL^−1^) and do not interfere with basal ROS that are needed for normal cell signaling and metabolic functions [22]. At a higher dose (400 µg mL^−1^) they induce reactive oxygen species (ROS) generation that mediates cellular apoptosis. It is clearly demonstrated that cell death is driven by the mitochondrial apoptotic pathway, wherein ROS-mediated mitochondrial membrane depolarization is followed by DNA damage and caspase cascade activation. To date, this is the first report on the mechanism of nanoceria toxicity in lens cells.

## 2. Results

### EGCNPs Characterization

**Figure 1:**
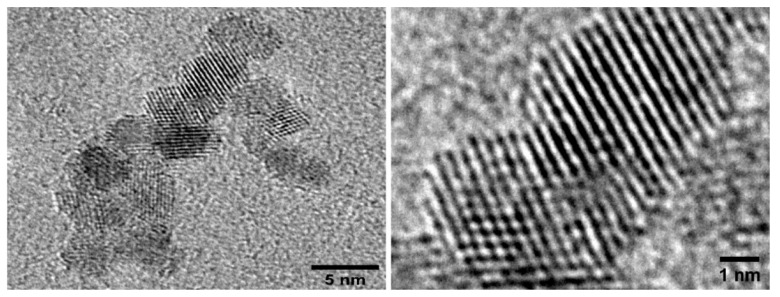
TEM micrographs of ethylene glycol coated nanoparticles EGCNPs showing the spherical, monodisperse and crystalline nature of the nanoparticles.

EGCNPs were thoroughly characterized in our previous work using transmission electron microscopy, powder X-ray diffraction, dynamic light scattering, thermogravimetric analysis, gas chromatography-mass spectroscopy, Fourier transform infrared spectroscopy (FTIR), UV-Vis spectroscopy [20]. Briefly, EGCNPs were spherical in shape with a core size (TEM) of 4.0 ± 0.8 nm (Figure 1) and crystallite size (XRD, Scherrer formula) of 3.5 nm. EGCNPs were stable in aqueous media for at least seven days with minimum sedimentation. The zeta potential was +44 mV in water and −9.7 mV in cell culture media due to protein coronae formation. EGCNPs were monodisperse with unimodal distribution of intensity-weighted mean hydrodynamic diameters as was demonstrated by DLS [20].

### Acute Exposure to High EGCNPs Concentrations Increases Basal ROS

**Figure 2:**
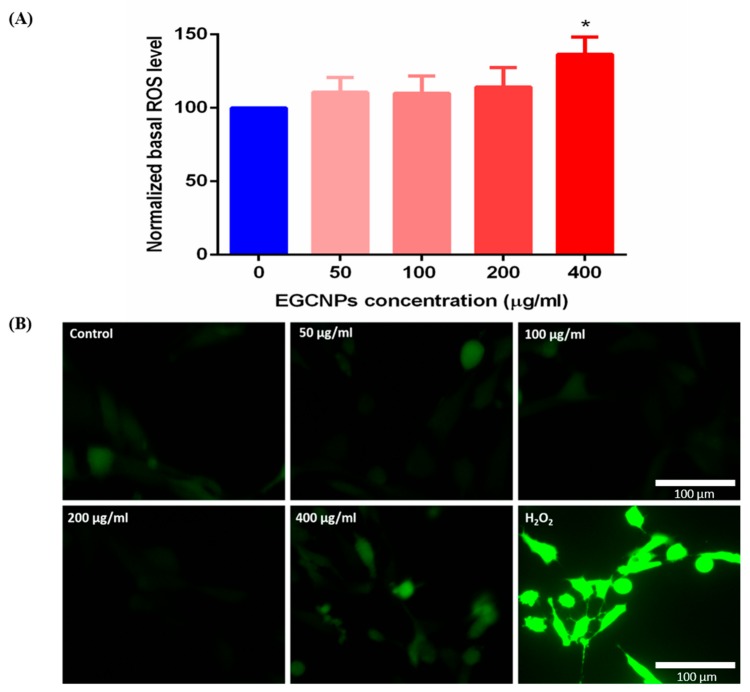
(**A**) Effect of EGCNPs (24 h exposure) on basal ROS level in HLECs measured by H_2_DCFDA fluorescent probe using a plate reader. The asterisk denotes statistical significance (*p* < 0.05) from negative control (0 µg mL^−1^), n ≥ 3 where n is the number of replicates using ANOVA followed by Dunnett’s multiple comparisons test. Error bars are presented as mean ± standard error of the mean (SEM) (**B**) Fluorescent microscope images after H_2_DCFDA staining of HLECs treated with different EGCNPs concentrations for 24 h. H_2_O_2_ (200 µM) was used as a positive control. Images were taken using a fluorescent microscope (Evos FL) using the same intensity power (20%) with minimal exposure duration to avoid auto-oxidation of the probe.

Overproduction of reactive oxygen species (ROS) is one of the most common pathways involved in nanomaterial toxicity [14]. Therefore, the basal ROS level in HLECs was measured after 24 h exposure to different EGCNPs concentrations (0, 50, 100, 200, and 400 µg mL^−1^) using the H2DCFDA probe. H2DCFDA is a non-fluorescent cell-permeable probe which is cellularly retained upon internalization due to deacetylation, and then oxidized by the action of ROS into highly fluorescent DCF enabling the estimation of ROS levels. As shown in Figure 2, EGCNPs concentrations up to 200 µg mL^−1^ did not lead to any significant alteration to the basal ROS level when compared to control cells (*p* > 0.05). Conversely, when the concentration was increased to 400 µg mL^−1^, a significant elevation in ROS level was observed.

### EGCNPs Localize in the Mitochondria

**Figure 3:**
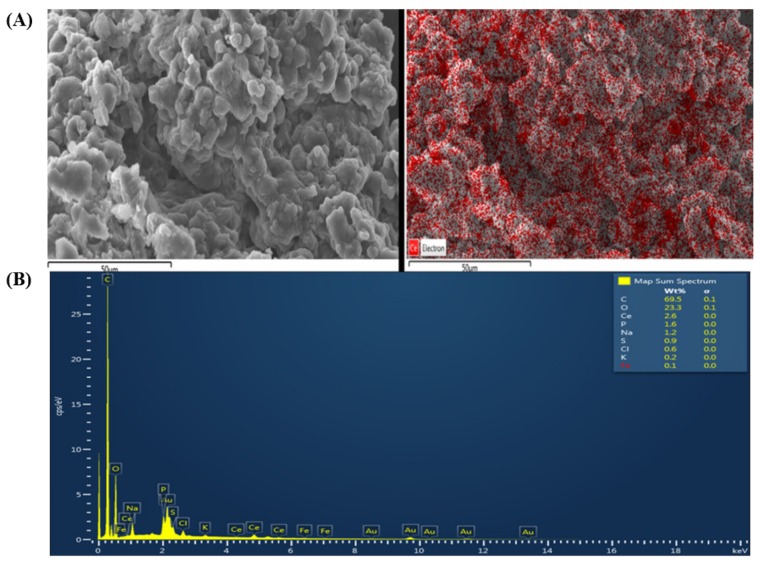
(**A**) SEM micrograph of the mitochondria isolated from HLECs treated with EGCNPs (left) and its associated cerium EDX mapping (right), (**B**) EDX spectrum and semiquantitative full elemental analysis generated from EDX mapping of the mitochondria. The presence of gold (Au) is due to sample coating with gold. Scale bar = 50 µm.

Since the mitochondria are the main source of ROS generation [14], it was necessary to investigate if EGCNPs exert their impact on ROS levels through their localization in the mitochondria. EGCNPs-treated HLECs were harvested and their mitochondria were isolated from the cytosolic fraction by differential centrifugation using a standard mitochondria isolation procedure [23]. The isolated mitochondria were then examined with a scanning electron microscope (SEM) and the presence of cerium was checked for using energy dispersive X-ray spectroscopy (EDX). EDX is a valuable tool enabling the identification of different elements based on their emitted characteristic X-rays after excitation with a high accelerating voltage electron beam [24]. Figure 3A shows an SEM micrograph of the isolated mitochondria (left) and its associated cerium EDX map (right) (the red regions are associated with high cerium characteristic X-ray emissions). The full elemental composition of the scanned map is displayed in Figure 3B and the Mα and Lα characteristic X-ray emission peaks for cerium were observed at 0.88 KeV and 4.83 KeV respectively. Furthermore, semi-quantitative EDX elemental analysis shows that cerium was the third most abundant element in the mitochondria following carbon and oxygen. These findings clearly confirm that significant localization of EGCNPs in the mitochondria occurs within 24 h of treatment.

### Effect of EGCNPs on the Mitochondrial Network

**Figure 4:**
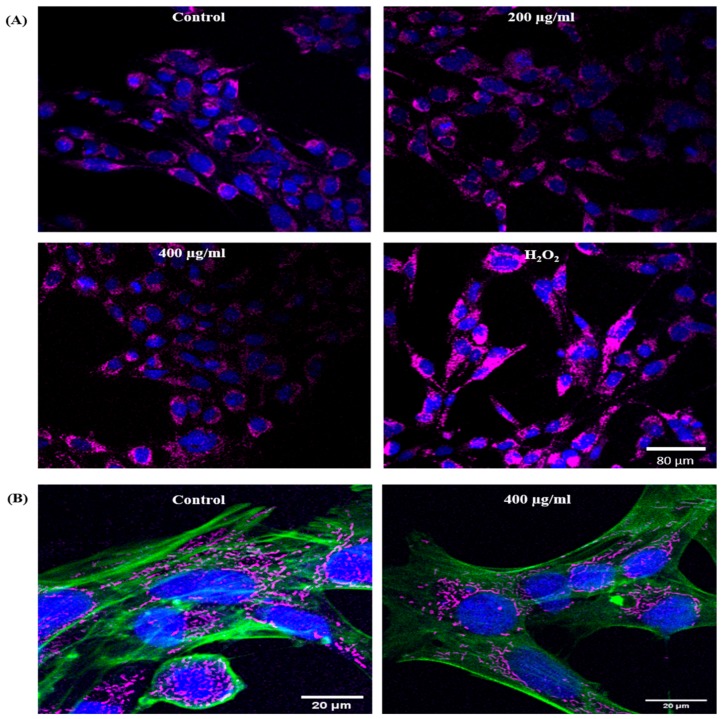
(**A**) Representative confocal images showing the effect of different EGCNPs concentrations (24 h exposure) on the mitochondrial morphology and organization (magenta) in HLECs, nuclei are stained with Hoechst 33,342 (blue) (**B**) High magnification confocal images of the mitochondria counterstained with cytoskeleton selective stain ActinGreen 488 (green) and Hoechst 33,342 (blue). No significant changes from control were observed up to EGCNPs concentrations of 400 µg mL^−1^. H_2_O_2_ (400 µM) was used as a positive control which shows significant aggregation of the mitochondria.

To examine the effect of EGCNPs on the mitochondrial morphology and network organization, staining with the mitochondria-selective stain (Mitotracker™ Red CMXRos) was employed. The mitochondria were uniform in shape and organization when treated with EGCNPs concentrations of up to 400 µg mL^−1^ and showed no significant difference from control cells (Figure 4A). The mitochondria were short and rod-shaped with organized localization in the perinuclear region (Figure 4B). H_2_O_2_ (positive control) caused significant mitochondrial aggregation and diffusion of the mitochondrial network was observed.

### EGCNPs Overdose Disrupts Mitochondrial Membrane Potential (∆Ψm)

**Figure 5:**
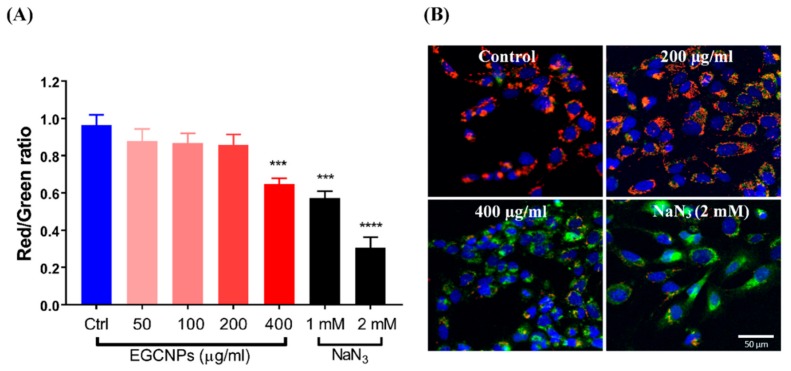
Effect of EGCNPs concentrations on mitochondrial membrane potential (∆Ψm) measured using JC-1 staining. Decrease in red/green ratio indicates depolarization of the mitochondrial membrane. (**A**) Quantification of red/green ratio from high throughput Incucyte S3 fluorescent images (images available in the SI), (**B**) Representative high magnification confocal microscopy images with JC-1 staining counterstained with Hoechst 33,342 (blue) showing significant increase in green/red ratio (depolarized ∆Ψm) at 400 µg mL^−1^. Up to 200 µg mL^−1^, the red and green had a ratio close to 1 causing them to colocalize. NaN_3_ was used as a positive control as it is a known disruptor of ∆Ψm. Asterisks denote statistical significance (***) *p* ≤ 0.001 and (****) *p* ≤ 0.0001 from control, n = 3, using one-way ANOVA, Dunnett’s multiple comparisons. Error bars are presented as mean ± SEM.

The integrity of the mitochondrial membrane potential is one of the most critical factors in assessing the function of the mitochondria; its depolarization (loss of normal charge distribution on both sides of the membrane) is an indicator for early stage apoptosis [25,26,27]. JC-1 dye was used to differentiate between healthy and depolarized mitochondria based on the change in the fluorescence of the dye. In healthy mitochondria, the cationic dye accumulates into the negatively charged interior of mitochondria where it forms J-aggregates shifting the fluorescence from green to red. Consequently, decreased red/green ratio is an indicator for mitochondrial membrane depolarization [28]. This ratio is highly accurate as it reports on changes in membrane potential regardless of the shape, size or density of the mitochondria [28]. As shown in Figure 5, EGCNPs concentrations up to 200 µg mL^−1^ had no significant effect on the (∆Ψm). When the concentration was increased to 400 µg mL^−1^, significant depolarization was observed suggesting that early stage apoptosis was taking place. For comparison, two different concentrations of sodium azide (NaN_3_) were used as positive controls since it is known to induce mitochondrial depolarization by blocking complex IV in the mitochondrial electron transport chain [28].

### ATP Level (Luciferase Assay)

**Figure 6:**
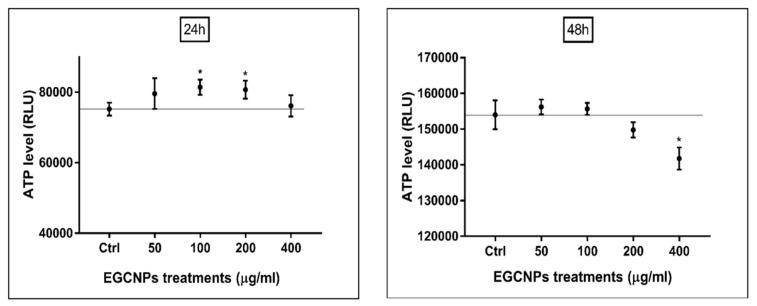
Effect of EGCNPs on ATP production in HLECs measured by luciferin-luciferase bioluminescent assay at 24 h and 48 h. Asterisks denote statistical significance from control (*p <* 0.05), n = 3 using one-way ANOVA. Error bars are presented as mean ± SEM.

The intracellular ATP production is considered a well-accepted differentiator between apoptotic and necrotic cell death [29,30]. Apoptosis is an energy-driven process that requires ATP for its execution such as in the breakdown of macromolecules [30]. When the ATP level drops significantly, it is an indication of the termination of controlled cell death and necrosis starts to take over. The changes in intracellular ATP production upon exposure to different EGCNPs concentrations were measured based on the bioluminescence produced using the highly sensitive luciferin-luciferase assay [29]. Up to 200 µg mL^−1^, no significant decline in ATP level was observed as expected from the aforementioned results (Figure 6). Interestingly, no significant change in ATP level was observed at 400 µg mL^−1^ even though mitochondrial membrane depolarization does take place at this concentration. The cells were then treated for a longer duration (48 h), and a significant decrease in ATP production was observed at 400 µg mL^−1^ (Figure 6). Another notable finding was the slight but significant increase in ATP levels at 100 and 200 µg mL^−1^ EGCNP concentrations after 24 h.

### Genotoxicity (pH2AX Immunocytochemistry Assay)

**Figure 7:**
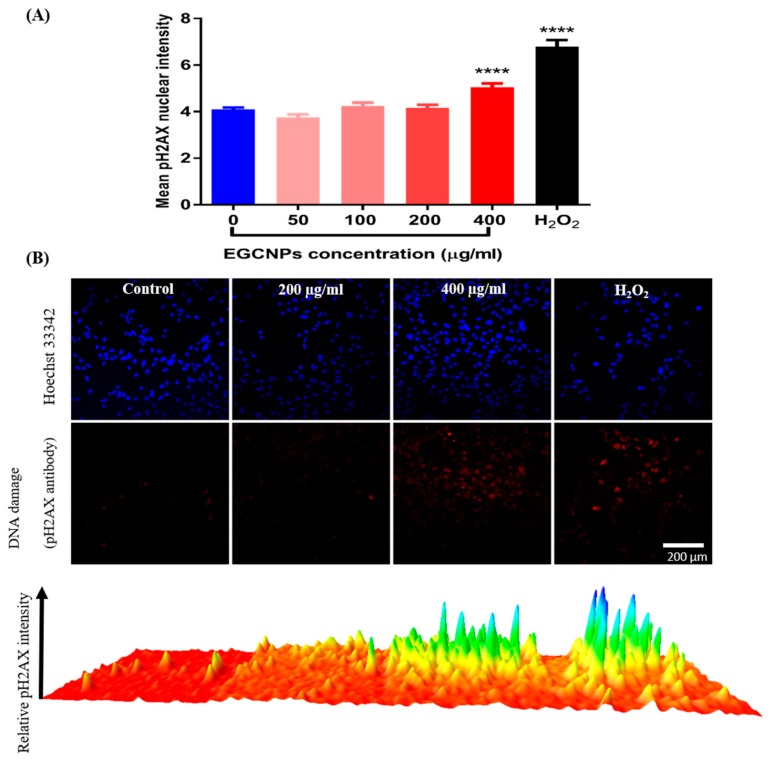
Effect of EGCNPs on DNA damage (genotoxicity) measured by immunocytochemistry thorough detection of pH2AX mean intensity. (**A**) quantification of pH2AX fluorescence intensity (only pH2AX signal inside nuclei perimeters) within at least 500 individual nuclei obtained from 4 independent experiments. (**B**) representative confocal images and 3D surface plotting of relative pH2AX intensity (red). Asterisks denote statistical significance from control (*p* < 0.05) using one-way ANOVA, Dunnett’s multiple comparisons. Error bars are presented as mean ± SEM. A schematic showing DNA damage quantification steps is shown in the Supplementary Information.

DNA integrity in HLECs is particularly important as its damage could lead to impaired protein synthesis, impaired differentiation and consequently cataract formation [31]. Since DNA is a common target of ROS [32], EGCNP induced genotoxicity was evaluated by detecting DNA double strands breaks (DSBs). As a response to DSBs, the histone protein H2AX is phosphorylated (pH2AX) and this can be detected with immunocytochemistry. The results in Figure 7 show that increased ROS was accompanied with increased DNA damage at 400 µg mL^−1^ whereas, at low concentrations, no genotoxicity was observed.

### Effect of EGCNPs on Caspase-3,7 Activity

**Figure 8:**
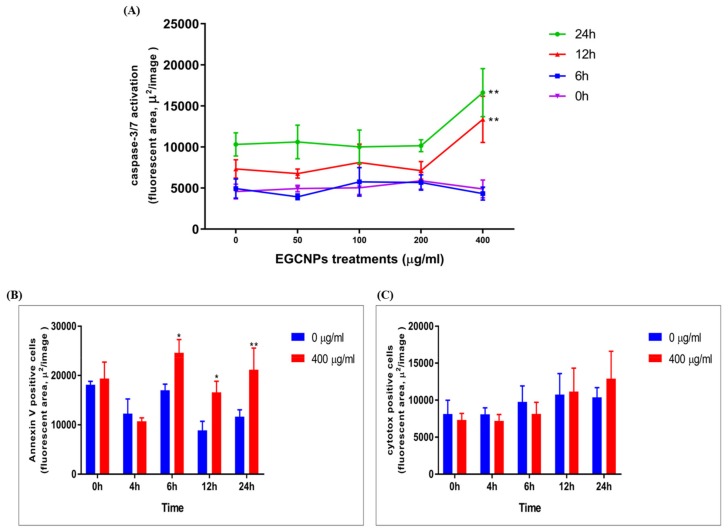
Effect of different EGCNPs concentrations on (**A**) caspase-3,7 activity, (**B**) Annexin V binding after 24 h, (**C**) Cytotox Red staining after 24 h in HLECs. No significant caspase activation was observed up to 200 µg mL^−1^. At 400 µg mL^−1^, significant caspase activity was observed after 12 h of treatment. Asterisks (*) p ≤ 0.05 and (**) p ≤ 0.01 denote statistical significance from control in same time group, n = 4 where n is the number of replicates, using two-way ANOVA. Error bars are presented as mean ± standard error of the mean (SEM). Representative images that were used for quantification are available in the Supplementary Information.

Activation of caspases is a key indicator of cells irrevocably submitting to apoptotic death [33]. The activation of caspases 3 and 7 (effector caspases in mammals) in HLECs upon EGCNPs treatment was detected over 24 h by means of live cell imaging using the IncuCyte^®^ Caspase-3/7 Red apoptosis assay reagent. The reagent consists of a red fluorophore (NucView™633) attached to an activated caspase recognition motif (DEVD). Upon binding with activated caspases, the motif is cleaved, and the fluorophore that is liberated intercalates with DNA giving red fluorescence proportional to caspase activity that can be quantified over time. Figure 8A shows that EGCNPs do not result in significant caspase activation up to concentrations of 200 µg mL^−1^ compared to control cells. Overexposure to EGCNPs (400 µg mL^−1^) caused significant caspase activation as early as 12 h from the treatment commencing. This agrees with the JC-1 data (Figure 5) where loss of membrane potential was observed only at 400 µg mL^−1^.

### Annexin V/Cytotox Red Assay

To confirm whether cells undergo apoptosis rather than necrosis at EGCNP concentration of 400 µg mL^−1^, the cells were double stained with annexin V green/Cytotox Red and observed by means of live cell imaging using Incucyte S3 live imaging system. Apoptotic cells only show annexin V labelling (green) while late apoptotic or necrotic cells are double labelled with both stains. Figure 8B shows that the 400 µg mL^−1^ concentration caused a significant increase in cells labelled with annexin V, when compared to negative control after treatment durations of 6 h, 12 hand 24 h. No significant change was found in the number of necrotic cells (labelled with Cytotox Red) over the same treatment durations (Figure 8C). These findings indicate that apoptosis is the main mechanism of cell death in HLECs with acute overexposure to EGCNPs.

## 3. Discussion

Cerium oxide nanoparticles have shown antioxidant activities in different mammalian cell lines because of their ability to scavenge for different ROS in an enzyme-mimetic fashion [4,17,34]. They have shown promise in the treatment of many ocular diseases such as retinal injury [17]. Additionally, we have recently reported that a novel ultra-small (4 nm) and well characterized monodisperse nanoceria formulation coated with modified ethylene glycol can protect HLECs from oxidative stress induced by H_2_O_2_ when used at concentrations as low as 50 µg mL^−1^, indicating that nanoceria may have potential as an anticataract treatment [20]. Interestingly, it was found that HLECs can proliferate normally with the same rate when treated with EGCNPs concentrations up to 400 µg mL^−1^ for three days. However, a significant decrease in viability measured by MTT assay was observed at 24 h suggesting that the mitochondria may be affected with nanoceria treatment since the MTT assay assesses the viability based on the activity of mitochondrial succinate dehydrogenase [35]. Consequently, in this study, the aim was to elucidate the biological consequences of the interaction between EGCNPs at 400 µg mL^−1^ and mitochondria in HLECs. Concentrations above 400 µg mL^−1^ caused a marked decrease in proliferation and cell membrane damage (necrosis) and were not subject to further study.

The overproduction of ROS is involved in the etiology and pathogenesis of many diseases and disorders such as Alzheimer’s, Parkinson’s, diabetes, and cataract. The identification and effects of new drugs on the redox state of a target cell line/cell/tissue is essential [36,37,38,39]. In this study, the basal ROS level in HLECs was not affected at low concentrations of EGCNPs. However, a significant increase in basal ROS was seen when the concentration was increased to 400 µg mL^−1^ (Figure 2). This shows that even though EGCNPs possess antioxidant and ROS scavenging properties, short-term overexposure can elevate ROS levels. This increase in ROS levels is likely to have been caused by Fenton like reactions where reduced metal ions (e.g. Ce^3+^) react with cellular hydrogen peroxide (H_2_O_2_), producing hydroxyl radicals (HO•) that are highly toxic to biomolecules [32]. It was previously shown that EGCNPs had surface trivalent cerium and hence the progression of a Fenton-like reaction is expected to progress as reported in the literature [40]. It is of significant importance that therapeutic doses of EGCNPs (< 200 µg mL^−1^) do not alter the basal ROS level which is needed to maintain normal metabolic functions and signaling pathways [22]. Our previous work showed that EGCNPs used at 50 µg mL^−1^, scavenge for ROS induced by H_2_O_2_ in HLECs. Considering the results shown here, this suggests that at low concentrations, EGCNPs only exert their ROS lowering capability in the presence of external ROS stimuli without diminishing the cellular benefits of basal ROS.

Since the mitochondria are the major sites for ROS generation [14], it was speculated that their properties and function would be altered with acute exposure to EGCNPs. EGCNPs have been shown to localize mainly in the cytoplasm of HLECs, however, organelle-specific localization was not identified [20]. In this study, the mitochondrial localization of EGCNPs was confirmed by examining isolated mitochondria from EGCNPs-treated HLECs using SEM-EDX (Figure 3). By using this technique, it was feasible to avoid surface functionalization with fluorescent markers; a common practice in the literature to track the nanoparticles that changes the surface properties and in turn could affect the uptake behavior of nanoparticles [41]. The mitochondrial localization of EGCNPs is likely to be caused by the strong positive zeta potential value that EGCNPs carry (+44 mV) making the negatively charged interior of the mitochondria an ideal target [36]. This finding is interesting since it is known from our previous investigation that protein coronae are adsorbed on the surface of EGCNPs making the zeta potential value slightly negative (−9.7 mV) in cell culture media containing fetal bovine serum. Nonetheless, the coronae do not appear to prevent the nanoparticles from entering the mitochondria. The zeta potential is known to play a significant role in subcellular localization of nanoceria which affects both the toxicity and activity profile [42]. It has previously been shown that targeting nanoceria specifically to mitochondria by surface functionalization with the mitochondrial-targeting cationic ligand (triphenylphosphonium, TPP), suppresses oxidative stress-induced neuronal death in mice [36]. Our findings indicate that this specific functionalization is not required for mitochondrial uptake in HLECs. Following the confirmation of mitochondrial uptake of EGCNPs, the effect of such uptake on the morphology and network organization of the mitochondria was studied using confocal microscopy and was found to be similar up to 400 µg mL^−1^ Figure 4). This suggested that at the higher concentration (400 µg mL^−1^) the cells could be undergoing early stage apoptosis, in which the shape of the mitochondria remains intact [25,30]. Hence, some of the key apoptotic hallmarks were further investigated.

One of the key indicators of early stage apoptosis is the loss of mitochondrial membrane potential where membrane depolarization and formation of mitochondrial membrane transition pores allow the release of mitochondrial intermembrane space enzymes (e.g. cytochrome c) that activate the caspase cascade initiating apoptosis [26,33]. Indeed, significant mitochondrial membrane depolarization at the higher dose of EGCNPs was observed and this indicated that apoptosis was taking place (Figure 5). To support these findings, the ATP level in EGCNPs treated HLECs was measured. Since apoptosis is an energy-dependent process, it was expected that the ATP level should remain relatively the same during the initial stages of apoptosis. Various studies have shown that ATP levels do not change during the initial stages of apoptosis [29]. This was the case in EGCNPs-treated HLECs as the 400 µg mL^−1^ concentration showed no significant difference in ATP level from control cells or those treated with lower concentrations of nanoceria (Figure 5). This could be explained by the fact that cells require ATP in order to initiate the controlled apoptotic cell death through various ATP-dependent steps such as caspase activation, nuclear condensation and apoptotic body formation [29]. To further test this assumption, the cells were treated for a longer duration (48 h), and indeed a small but significant decrease, in ATP at 400 µg mL^−1^ was observed, indicating the termination of apoptosis in some of the cells with concomitant ATP depletion. It is noteworthy that at 24 h, safe EGCNPs concentrations (≤ 200 µg mL^−1^) caused a slight increase in ATP levels. One possible reason is that the uptake of EGCNPs may have proceeded through energy-dependent clathrin-mediated and caveolae-mediated endocytic pathways, which have been reported as the main uptake mechanism of nanoceria in many cell lines [41,42,43].

DNA integrity is of particular interest in HLECs since the cells are continually synthesizing lens proteins (crystallins), and hence damage could lead to disrupted protein expression and eventually lead to cataract formation [44]. It was shown before that nanoceria coated with 3-phosphonopropionic acid have no adverse effect on DNA health when exposed to a concentration of 10 µg mL^−1^ for 24 h [45]. It was demonstrated that EGCNPs can be tolerated at a 20-fold higher concentration (200 µg mL^−1^) for the same treatment duration. However, a dose of 400 µg mL^−1^ caused significant DNA double strand breaks (as shown in Figure 7). DNA damage is likely to be caused by increased ROS that attack the DNA, causing double strand breaks. It was reported that some nuclear uptake of EGCNPs take place at therapeutic concentrations (less 200 µg mL^−1^) [20]. This nuclear uptake is likely to have been increased at 400 µg mL^−1^ causing EGCNPs to aggregate inside the nuclei promoting genotoxicity [16]. Even though some nanoparticles have poor permeability into the nuclei, their entry is inevitable during mitosis where the nuclear membrane is broken as the cells prepare for division [46]. As such, higher doses of EGCNPs will provide more opportunity for increased localization and possibly aggregation into the nuclei. The aforementioned events are likely to have led to the activation of caspases at (400 µg mL^−1^) which are the key causes of apoptosis (Figure 8) [33]. Additionally, annexin V/Cytotox Red assay conclusively demonstrated that apoptosis is the main driver for cell death, where an increase in annexin V binding was observed without concomitant increase necrosis-specific Cytotox Red labelling (Figure 8). Together these results demonstrate that the main mechanism of cerium oxide toxicity in HLECs proceeds through a mitochondrial apoptotic pathway mediated by elevated ROS and DNA damage.

The toxicity of nanoceria has been studied in vitro using a range of cell lines (reviewed in Gagnon and Fromm, 2015) but the data are not comparable given the varying characteristics of nanoceria used including the shape, size, colloidal stability, zeta potential, surface valence, surface functionality and the range of nanoceria fabrication methods. There are still inconsistencies in the literature regarding the mechanism of nanoceria toxicity [13]. Our results show, in a dose-dependent manner, the toxicity mechanism of well characterized nanoceria (EGCNPs) in human lens epithelium. To conclude, when considering nanoceria toxicity, and given their redox active characteristics, emphasis must be on the evaluation of the mitochondrial functionality as an early indicator for cell health as it can reveal significant effects that can be easily overlooked in short-term proliferation and standard toxicological studies.

## 4. Materials and Methods

### Synthesis of Ethylene Glycol-Coated Cerium Oxide Nanoparticles (EGCNPs)

Cerium oxide nanoparticles coated with ethylene glycol and ethylene glycol mono- and di- acetates (EGCNPs) were synthesized in aqueous conditions using the ammonia precipitation method as reported previously [20].

### Cell Culture

Human lens epithelial cells (HLECs) (B3, ATCC^®^ CRL11421™) were cultured in Eagle’s minimum essential media (EMEM) (ATCC^®^ 30–2003) supplemented with fetal bovine serum (20%, Scientific Lab Supplies), penicillin (100 units/mL, Sigma Aldrich) and streptomycin (0.1 mg/mL, Sigma Aldrich). The cells were incubated at 37 °C and 5% CO_2_ in a humidified environment (95% RH). The experiments were carried out on HLECs in the log growth phase.

### Basal ROS Level

H_2_DCFDA probe (2′,7′-dichlorodihydrofluorescein diacetate, D399, ThermoFisher) was employed to measure basal ROS levels as previously reported [20]. Briefly, HLECs were seeded in 96-well plates in complete EMEM (5000 cells/well) and allowed to recover for 24 h. The media were then removed and replaced with fresh media containing different EGCNPs concentrations (0, 50, 100, 200, and 400 µg mL^−1^) for 24 h. A maximum of 400 µg mL^−1^ was previously reported as the highest concentration beyond which a marked decrease in proliferation is observed [20]. H_2_O_2_ (200 µM, Sigma Aldrich) was used as a positive control. After the treatment period, the media were discarded, cells were washed once with pre-warmed PBS (ATCC^®^ 30-2200™) and incubated with H2DCFDA solution (10 µM in PBS) for 30 min at 37 °C. H2DCFDA solution was then discarded, cell washed with pre-warmed PBS and fresh PBS (200 μL) was added to each well. DCF fluorescence intensity was measured using a microplate reader (FLUOstar Omega, BMG LABTECH, Ortenberg, Germany) at excitation/emission of 485/520 nm. Correcting the fluorescence values was performed before analysis by subtracting the fluorescence of unstained cells from all other values. No fluorescence was observed for cell-free H2DCFDA/PBS and H2DCFDA/PBS/EGCNPs mixtures.

### EGCNPs Mitochondrial Uptake and Localization (SEM-EDX Studies)

HLECs were grown in five T175 flasks until they reached approximately 80% confluence. The cells were then treated with EGCNPs (400 µg mL^−1^) for 24 h and subsequently harvested, pelleted and washed once with full media and once with PBS. The mitochondria were then isolated from the pellet using the Mitochondria Isolation Kit for Cultured Cells (89874, ThermoFisher, Paisley, UK) in accordance with the supplier’s instructions. The isolated mitochondria were washed once with PBS, fixed with paraformaldehyde (4%, 5 min) and dried in pure ethanol (5 min). The mitochondria were placed on a SEM aluminum stub covered with a carbon tape and the specimens were coated with a gold layer (5 nm) using a sputter coater (Q150R ES, Quorum Technologies Ltd, East Sussex, UK). The specimens were examined using a scanning electron microscope equipped with an EDX detector (JEOL, JSM-7100f, Tokyo, Japan). EDX spectra and mapping were acquired and processed using Aztec software (v.2011, Oxford Instruments, Abingdon, UK).

### Mitochondrial Morphology (Confocal Studies)

HLECs were seeded (5000 cells/well) in black 96-well plates with clear flat bottom (Falcon^®^ 353219, Corning, New York, US) and left to establish for 24 h. The following day, the media were discarded, and the cells were treated with EGCNPs-containing media (0, 50, 100, 200 and 400 µg mL^−1^) for 24 h. After the treatment period, the media were removed, and the cells were washed once with pre-warmed PBS. The cells were then incubated with full media containing MitoTracker™ Red CMXRos (200 nM, ThermoFisher, Paisley, UK) for 20 min at 37 °C. After staining, the cells were washed once with pre-warmed PBS, fixed in pre-warmed paraformaldehyde solution (4% in PBS) for 10 min at room temperature and permeabilized by incubating in ice-cold acetone for 5 min. The cells were then counterstained by incubating in PBS containing Hoechst 33,342 (2 μg/mL, ThermoFisher, Paisley, UK) for 20 min at room temperature. Imaging was carried out using a confocal laser scanning microscope (Leica, Wetzlar, Germany) using the following settings: sequential scanning, 20x dry objective, ex/em: Mitotracker: 543/599 nm, Hoechst: 405/461 nm.

### Mitochondrial Membrane Potential (JC-1 Staining)

JC-1 probe (Abcam) was employed for mitochondrial membrane potential measurements. HLECs were seeded, established and treated with different EGCNPs concentrations (0, 50, 100, 200 and 400 µg mL^−1^) as before and sodium azide (1 and 2 mM, Sigma Aldrich, Gillingham, UK) was used as a positive control. After the treatment period, the media were removed, and cells incubated with JC-1 probe in complete media (10 μM) for 30 min at 37 °C in dark conditions. The staining solution was removed, cells washed with PBS and replaced with phenol red-free complete media. The wells were then imaged using Incucyte S3 high throughput imaging system using the green and red channels (5 wells per treatment condition, three images per well) and quantification of the red/green ratio was calculated using the integrated software by dividing the fluorescence area of the red channel over the fluorescence area of the green channel. Other software (e.g. ImageJ (version 2, NIH, Maryland, US) can be employed for such calculations by thresholding the red and green fluorescence and subsequently calculating their corresponding areas. The experiment was run in triplicate. Qualitative high magnification images were acquired using a confocal microscope after nuclear staining with Hoechst 33342. The excitations/emission settings were as follows: Hoechst 33,342 (405/461 nm), JC-1 green monomers (488/530 nm), JC-1 red aggregates (543/590 nm).

### ATP Quantification (Luciferase Assay)

The quantification of cellular ATP was carried out using the luminescent luciferase assay (CellTiter-Glo, Promega) in accordance with the supplier’s instructions. Briefly, HLECs were established in 96-well plates as before and treated with different EGCNPs concentrations for 24 h and 48 h. After the treatment period, the media were removed and replaced with fresh media. An equal volume of CellTiter-Glo reagent was added to each well to lyse the cells and release a luminescent signal proportional to the amount of ATP present. The plate was placed on an orbital shaker for 2 min and then incubated in the dark at room temperature for 10 min to stabilize the signal. The luminescent signal was read using a luminometer (Infinite^®^ 200 PRO, TECAN, Männedorf, Switzerland).

### Genotoxicity (Immunocytochemistry)

The DNA damage was evaluated using the HCS DNA kit (ThermoFisher) according to the supplier’s instructions. Briefly, HLECs were seeded in a black 96-well plate, established and treated for 24 h with different EGCNPs concentrations (0, 50, 100, 200 and 400 µg mL^−1^) as before. After the treatment period, the media was removed, the cells were fixed in paraformaldehyde solution (4%, 10 min), rinsed with PBS, and permeabilized in 0.2% Triton-X-100 (15 min). The cells were washed with PBS and then incubated in a blocking buffer (bovine serum albumin in PBS, 1%) for 1 h at room temperature. The blocking buffer was removed, and the cells were incubated with the primary antibody solution (pH2AX mouse monoclonal antibody) for 1 h. The primary antibody solution was then removed, and the cells were washed three times with PBS and incubated with the secondary antibody/nuclear stain solution (Alexa Fluor 555 goat anti-mouse IgG/Hoechst 33342) for 1 h. The secondary antibody solution was then removed, the cells were washed three times with PBS, and replaced with fresh PBS before proceeding to imaging. Imaging was carried out using a confocal laser scanning microscope using the following settings (sequential scanning ex/em: pH2AX: 543/565 nm, Hoechst: 405/461 nm). For data analysis, Hoechst staining was used for nuclear segmentation and the DNA damage was measured by the increase in the fluorescence of pH2AX signal in the region defined as the nuclei. The experiment was repeated four times and at least 100 nuclei were analyzed in each replicate.

### Caspase-3,7 Assay (Live Cell Imaging)

HLECs were seeded in 96-well plates (5000 cells per well) and left to establish for 24 h. The cells were then treated with different EGCNPs concentration (0, 50, 100, 200 and 400 µg mL^−1^) in supplemented EMEM in presence of IncuCyte^®^ Caspase-3/7 Red Apoptosis Assay Reagent (0.5 µM, excitation/emission 630/650 nm). The cells were imaged every 2 h for 24 h using the Incucyte S3 live cell imaging system (Essen BioScience, Welwyn Garden City, UK) fitted inside an incubator (5% CO_2,_ 37 °C). Three fields of view were imaged per well using the 20× objective. Images were then analyzed, and caspase activity was expressed as the area of red fluorescent caspase signal per image. The experiment was performed four times.

### Annexin V/Cytotox Red Assay (Live Cell Imaging)

The assay was performed on the Incucyte S3 live cell imaging system as described above but in the presence of double staining IncuCyte^®^ Annexin V Green Reagent/IncuCyte™ Cytotox Red Reagent (250 nM). Excitation/emission maxima for Annexin V and Cytotox Red are 490/515 nm and 612/631 nm respectively.

## 5. Conclusions

The antioxidant properties of nanoceria are dose dependent with oxidative actions triggered if the concentration is increased beyond a certain threshold. Nanoceria manifest no cytotoxic or genotoxic effects in human lens epithelial cells when concentrations are up to 200 µg mL^−1^. At 400 µg mL^−1^, these nanoparticles induced ROS generation which mediated cellular apoptosis driven by the mitochondrial apoptotic pathway. Potential biomedical applications need to take this threshold into consideration. Further work is needed to determine thresholds for other cell and tissue types.

## Supplementary Materials

The following are available online at https://www.mdpi.com/1420-3049/25/3/441/s1, Figure S1: Representative Incucyte images used for the quantification of JC-1 red/green ratio in HLECs after treatment with different EGCNPs concentrations, Figure S2: Representative Incucyte images used for measurement of caspase-3,7 activity in HLECs after treatment with different EGCNPs concentrations (0–400 µg mL^−1^) for 24 h, Figure S3: Representative Incucyte images of Annexin V green/Cytotox Red staining in HLECs comparing control and EGCNPs treated cells (24 h), Figure S4: Schematic representation of the steps employed for the quantification of DNA damage.
