# Supplementary material for: Treatment of Human Lens Epithelium with High Levels of Nanoceria Leads to Reactive Oxygen Species Mediated Apoptosis
Source: Molecules. 2020 Jan 21;25(3):441. doi: 10.3390/molecules25030441 (PMC7036910; doi:10.3390/molecules25030441)
Supplement: Supplementary file 1 [file molecules-25-00441-s001.pdf]

Supporting Information

Treatment of human lens epithelium with higher levels of nanoceria leads to reactive oxygen species mediated apoptosis

Belal I. Hanafy,[a] Gareth W. V. Cave,[a] Yvonne Barnett,[a,b] and Barbara Pierscionek.[a,*]

[a] *School of Science and Technology, Nottingham Trent University, Clifton Lane, Nottingham NG11 8NS, UK.*

[b] *Faculty of Science and Technology, Anglia Ruskin University, East Road, Cambridgeshire CB1 1PT.*

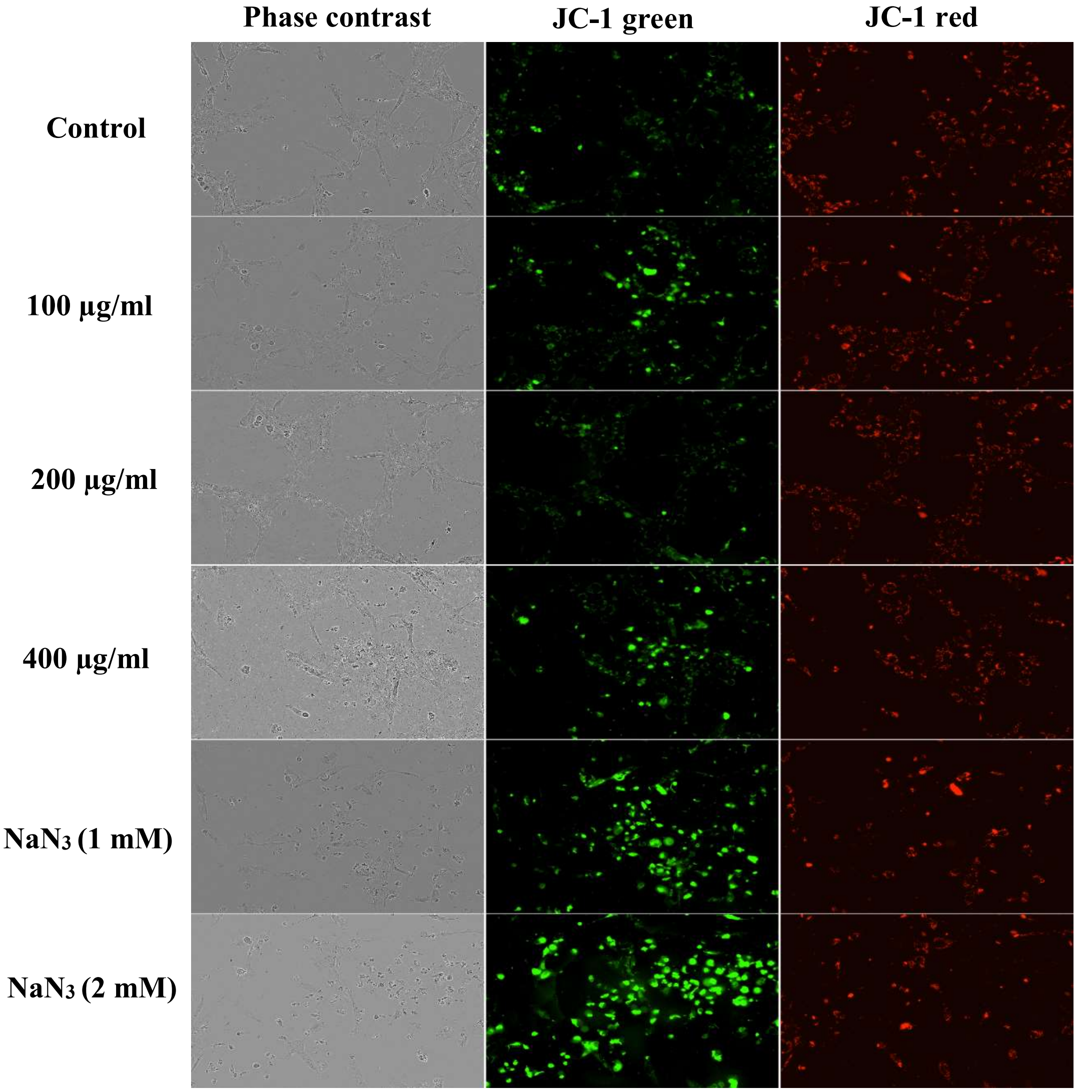

Figure S1 Representative Incucyte images used for the quantification of JC-1 red/green ratio in HLECs after treatment with different EGCNPs concentrations. Three fields of view were imaged in each well (in 96 well plate), at least four wells were used for each treatment condition. The experiment was independently repeated three times.

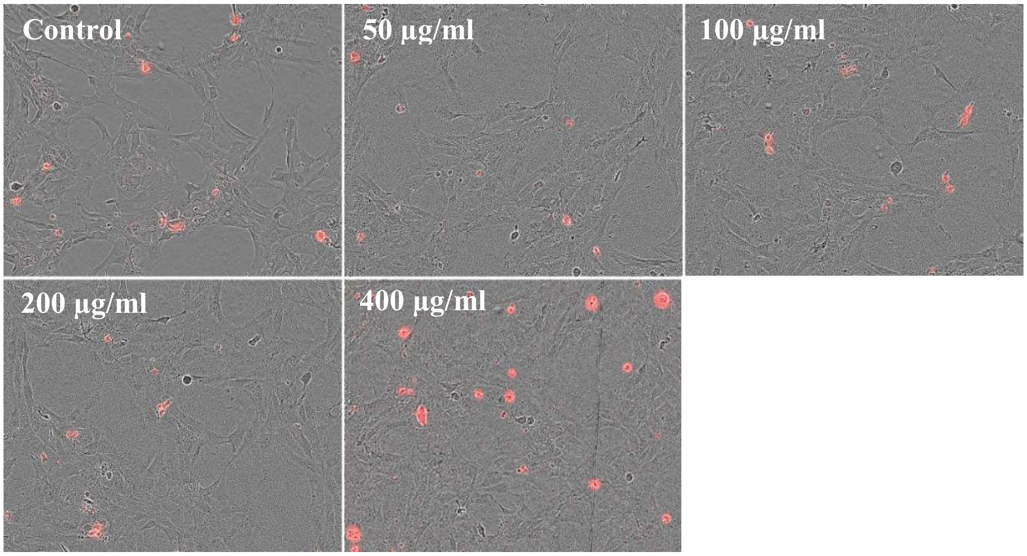

Figure S2 Representative Incucyte images used for measurement of caspase-3,7 activity in HLECs after treatment with different EGCNPs concentrations (0 - 400 µg ml^{-1}) for 24 hr. Three fields of view were imaged in each well (in 96 well plate), n=4

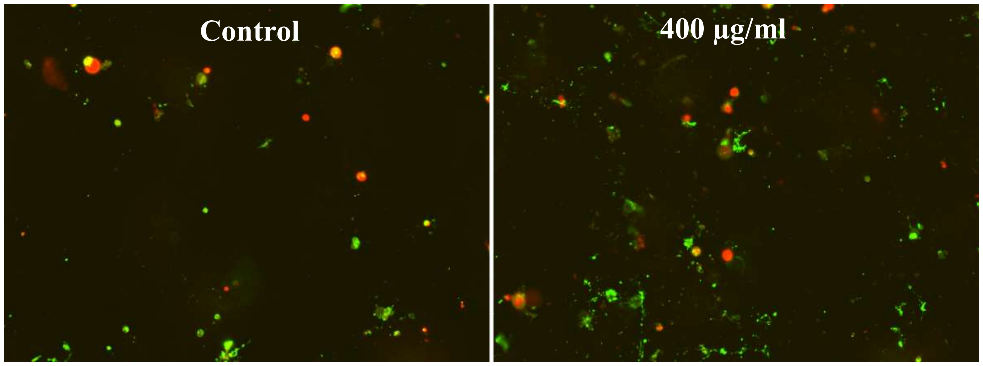

Figure S3 Representative Incucyte images of Annexin V green/Cytotox Red staining in HLECs comparing control and EGCNPs treated cells (24 h). Treated cells showed marked increase in annexin V labelling (apoptotic cells) without increase in number of necrotic cells (Cytotox Red).

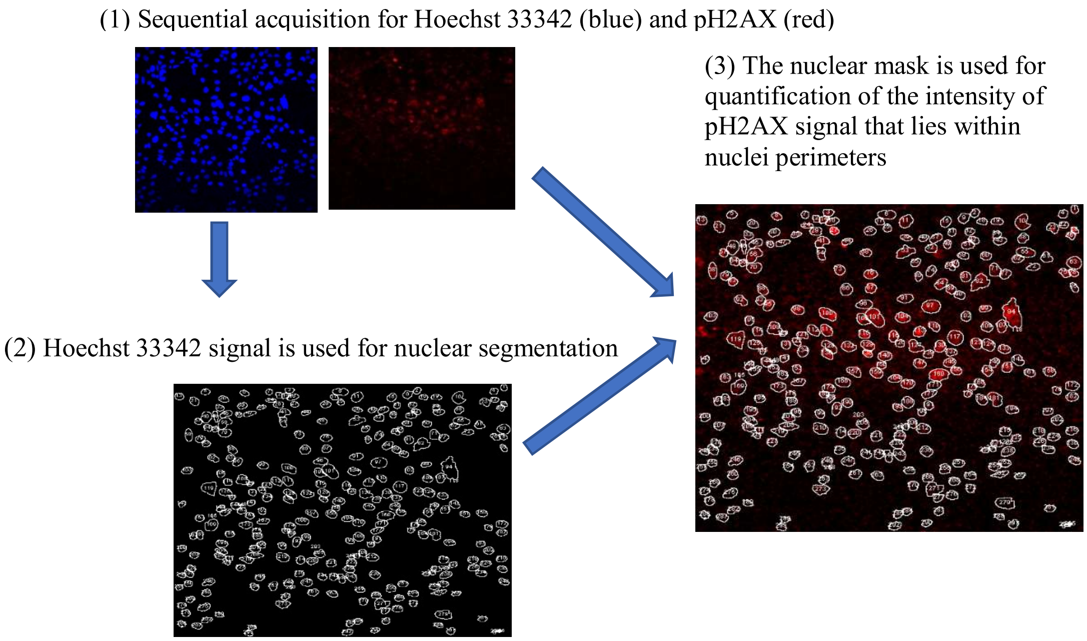

Figure S4 Schematic representation of the steps employed for the quantification of DNA damage (genotoxicity).
